# Editorial: Plant root interaction with associated microbiomes to improve plant resiliency and crop biodiversity, volume II

**DOI:** 10.3389/fpls.2023.1143657

**Published:** 2023-02-14

**Authors:** E. Malusà, N. Vassilev, D. Neri, X. Xu

**Affiliations:** ^1^ Department of Plant Protection, National Institute of Horticultural Research, Skierniewice, Poland; ^2^ Council for Agricultural Research and Economics - Center for Viticulture and Enology, Conegliano, Italy; ^3^ Department of Chemical Engineering, Institute of Biotechnology, University of Granada, Granada, Spain; ^4^ Dipartimento di Scienze Agrarie, Alimentari ed Ambientali, Università Politecnica delle Marche, Ancona, Italy; ^5^ NIAB, West Malling, United Kingdom

**Keywords:** biofertilizers, biopesticides, plant-soil-microbiome interactions, soil sickness, bioinocula safety, rhizobium symbiosis

Soil is acknowledged as a limited resource, which determines crop productivity and ecosystem sustainability. Microorganisms form an integral part of soil, contributing to soil fertility and plant health. The soil, diverse microbial communities and plants are involved in direct and indirect interactions, which are often difficult to predict, depending on environmental factors (Fierer, 2017). A comprehensive understanding of mechanisms underlying these interactions will enable development and implementation of strategies optimizing crop/soil management to improve crop resilience against biotic and abiotic stresses. The study of plant–soil relations is also considered fundamental to explain the vegetation dynamics that influence ecosystem composition and functioning. This can enhance understanding of ecological evolution and allows better prediction and mitigation of consequences of human-induced global changes, promoting a sustainable provision of ecosystem services (van der Putten et al., 2013).

Soil sickness is a widespread problem associated with replanting fruit crops and monocropping, with a complex etiology, and influenced by soil and climatic conditions (Monaci et al., 2017). In addition to nutrient availability and plant pathogens, recent evidence indicates potential roles played by litter autotoxicity related to the microbial activities and the exposure to fragmented self-DNA (Mazzoleni et al., 2015) in soil sickness. The severity of soil sickness and its symptom expression is significantly affected by complex interaction between soil management, location, root system, soil organic matter content (Polverigiani et al., 2018) in which soil microbiome plays a critical role. Thus, identifying agricultural practices that promote microbial activities become a key approach to overcome soil sickness. Such management practices include accelerating degradation of allelopathic compounds (Endeshaw et al., 2015), crop rotation, integrating weed management with living mulches (Mia et al., 2021; Neri et al., 2021), and applying different kinds of microbial-based or microbial-derived products (Vassileva et al., 2020).

To advance beyond a simplified view of individual plant-microbe or soil-plant interactions, the plants-soil biota should be considered as a unique “meta-organism”, influencing complex interactions that impact plant development and productivity. To facilitate efficient exploitation of beneficial microbial strains in practice, a complex set of data needs to be generated to assess their impact on resident soil microbiome (and its functions), soil physio-chemical properties and plant performance under the influence of agronomical practices (Malusà et al., 2021). This overarching view of plant-soil microbiota interactions in relation to crop performance underpins the Research Topic, which contains 11 contributions of reviews and original research articles.

Large-scale production and formulation of microbial strains, alone or in consortia, are the critical steps in the development of commercial products (Vassilev et al., 2017; Vassilev et al., 2020; Vassileva et al., 2022). Applying products from co-formulation of two or more beneficial microbes with complementary functions resulted in better wheat plant growth/health and more diverse and abundant beneficial endophytic bacterial communities in the root system (Zhang et al.), as also found in other crops. Lignocellulose-decomposing enzymes produced and released by the studied microorganisms were shown to have played a crucial role in the assembly of endophytic bacterial communities and their successful colonization of wheat roots. Stimulation of plant metabolic activities by biofertilizers was reviewed by Chaudhary et al., highlighting the multifunctional potential of such bioinocula in terms of promoting growth and activating plant defense in the presence of biotic and abiotic, particularly climate related, stresses. Inoculation of cuttings of the medicinal plant *Bacopa monnieri* with *Bacillus subtilis* and *Klebsiella aerogenes* individually or in combination induced a significant improvement in the accumulation of bacoside A, a triterpenoid saponin with preventive activity against Alzheimer’s disease (Shukla et al.), demonstrating the potential of the *in planta* production of secondary metabolites for medical applications.

Among environmental stresses, acidification is considered to limit soil fertility and health. Li et al. provided a novel insight into the relationship between lime amendment of acidified soil, composition of citrus root-associated microbiota and citrus tolerance to Huanglongbing (a systemic soil-borne disease caused by nonculturable bacteria *Candidatus Liberibacter* spp.). Liming, increased the diversity of root bacterial communities and enriched beneficial microorganisms in roots, two features associated with a lower Huanglongbing disease severity.

Soil fertility and crop productivity are closely related and dependent on soil biodiversity. Alternative strategies to increase crop diversification can supply several ecosystem services related to nutrient cycling, crop productivity and health, all mediated through soil microbiota. Trinchera et al. studied five cropping systems of organic vegetables and showed that multi-cropping increased the total soil microbial mass, promoting specific bacterial and fungal phyla and mycorrhizal colonization. These changes were associated with positive effects on C and P nutrient cycles and pathogen reduction. Multicropping was successfully applied in greenhouse production of *Capsicum annum* intercropped with *Foeniculum vulgare* (Yang et al.). The release of antimicrobial terpene compounds by the fennel roots suppressed *Phytophthora* disease through the induced accumulation of reactive oxygen species in the pathogen.

Biocontrol with specific microbial strains is an efficient tool for achieving sustainable pest control in an environmentally friendly manner. El-Saadony et al. reviewed literatures providing evidence of biocontrol activity of plant growth promoting microorganisms. Exploiting this multifunctional capacity can foster alternative strategies of crop protection, currently still relying on synthetic fungicides, thus responding to the challenge of ensuring healthy and fresh products within a net-zero horticulture (Xu, 2022). Duan et al. isolated a *Bacillus licheniformis* strain from apple roots, characterized its metabolic activity, particularly the degradation of phlorizin [a phenolic compound associated to apple replant disease (ARD)], identified possible mechanisms of action against several soil-borne pathogens, and optimized fermentation process for the large-scale production. Detailed studies about the effect of this strain on plant root development, soil-borne pathogens and soil microbial community led them to propose a framework for managing ARD. In another study (Alwahshi et al.), *Streptomyces violaceoruber* UAE1 isolated from rhizospheric soil of healthy date palms, able to produce 1-aminocyclopropane-1-carboxylic acid deaminase, showed antifungal activities against *Fusarium solani*, the causal agent of the sudden decline syndrome of date palm, highlighting the potential of this strain in disease management.

Belleís-Sancho et al. studied the relationship between gene expression induced by a beta-rhizobial *nifA* mutant (*Paraburkholderia phymatum*, a model organism for studying beta-rhizobia-legume symbiosis) and auxin synthesis during the symbiosis with *Phaseolus vulgaris*. In general, the establishment of this symbiotic interaction relies on a sophisticated molecular and chemical cross-talk between *Rhizobium* and the plant. The gene regulating nitrogenase expression, *nifA*, was shown to control expression of two bacterial genes involved in auxin synthesis, leading to their increased production in nodules occupied by the *nifA* mutant compared to wild-type nodules. It was concluded that the increased abundance of rhizobial auxin in the non-fixing *nifA* mutant strain may have played a role in root infection and early-stage symbiotic interactions.

Safety and quality are important issues for commercialization of biofertilisers and biopesticides. Vassileva et al. reviewed the risks of contamination of microorganism-bearing fertilizers during different production stages, storage, and application in soil. Direct and indirect spread of zooptic and plant pathogens associated with these microbial-based products were discussed with examples of different products, including compost, manure, biosolids, and bioformulated inoculants with plant growth promoting or biocontrol functions.

The work published in this issue confirms the urgent need for studies on soil microorganism-mediated processes responsible for different soil functions and ecosystem services, with the emphasis on their regulating mechanisms. This knowledge can be used to inform advisors and farmers and improve their decision-making in crop management to improve food security and quality (Borsotto et al., 2022).

## Author contributions

All authors listed have made a substantial, direct, and intellectual contribution to the work and approved it for publication.
